# Indicators of Child Health, Service Utilization and Mortality in Zhejiang Province of China, 1998–2011

**DOI:** 10.1371/journal.pone.0062854

**Published:** 2013-04-25

**Authors:** Wei Fang Zhang, Yan Hua Xu, Ru Lai Yang, Zheng Yan Zhao

**Affiliations:** 1 Department of Disease Screening, the Children’s Hospital, Zhejiang University School of Medicine, Hangzhou, China; 2 Key Laboratory of Reproductive Genetics (Zhejiang University), Ministry of Education, Hangzhou, China; Old Dominion University, United States of America

## Abstract

**Objective:**

To investigate the levels of primary health care services for children and their changes in Zhejiang Province, China from 1998 to 2011.

**Methods:**

The data were drawn from Zhejiang maternal and child health statistics collected under the supervision of the Health Bureau of Zhejiang Province. Primary health care coverage, hospital deliveries, low birth weight, postnatal visits, breastfeeding, underweight, early neonatal (<7 days) mortality, neonatal mortality, infant mortality and under-5 mortality were investigated.

**Results:**

The coverage rates for children under 3 years old and children under 7 years old increased in the last 14 years. The hospital delivery rate was high during the study period, and the overall difference narrowed. There was a significant difference (P<0.001) between the prevalence of low birth weight in 1998 (2.03%) and the prevalence in 2011 (2.71%). The increase in low birth weight was more significant in urban areas than in rural areas. The postnatal visit rate increased from 95.00% to 98.45% with a significant difference (P<0.001). The breastfeeding rate was the highest in 2004 at 74.79% and lowest in 2008 at 53.86%. The prevalence of underweight in children under 5 years old decreased from 1.63% to 0.65%, and the prevalence was higher in rural areas. The early neonatal, neonatal, infant and under-5 mortality rates decreased from 6.66‰, 8.67‰, 11.99‰ and 15.28‰ to 1.69‰, 2.36‰, 3.89‰ and 5.42‰, respectively (P<0.001). The mortality rates in rural areas were slightly higher than those in urban areas each year, and the mortality rates were lower in Ningbo, Wenzhou, and Jiaxing regions and higher in Quzhou and Lishui regions.

**Conclusion:**

Primary health care services for children in Zhejiang Province improved from 1998 to 2011. Continued high rates of low birth weight in urban areas and mortality in rural areas may be addressed with improvements in health awareness and medical technology.

## Introduction

In 2000, 189 heads of governments and at least 23 international organizations signed the Millennium Declaration, committing to achieve eight goals for health and development. Millennium Development Goal (MDG) 4 was to reduce under-5 mortality by two-thirds between 1990 and 2015. [1] There are only 3 years remaining to achieve this goal. Child mortality is declining in almost all countries. However, progress in reducing child mortality was substantially slower than the targeted annual rate of decline (4.4%) in some countries, especially developing countries. [2], [3] Some children die because of malnutrition, lack of primary care and poor infrastructure that could be avoided.

China, the largest developing country, has experienced dramatic social and economic developments since the reforms in the 1980s. The government was actively involved in the MDGs project. Increasing attention has been focused on monitoring and reducing maternal mortality, neonatal mortality, and mortality of children under-5 years old. In addition, a series of laws and regulations aiming to improve child health, education, law enforcement and the environment were implemented, including the 1994 Maternal and Infant Health Care Law of the People’s Republic of China [4] and the 1995, 2001, and 2011 Guidelines for Chinese Children’s Development [5], [6]. The Maternal and Infant Health Care Law governs postpartum visits, newborn screening, and primary health care services (e.g., regular physical examinations and vaccination) through the “Child Health Handbook”. [4] The main child health goals of the Guidelines for Chinese Children’s Development (2011–2020) are reducing infant mortality and under-5 mortality to 10 and 13 per 1000, respectively. The goals also include reducing disability, promoting the national immunization program (vaccination), and encouraging exclusive breastfeeding, reducing the incidence of low birth weight and reducing the prevalence of anemia. [5], [6] Zhejiang Province, one of the most affluent provinces on the eastern coast of China, includes 11 administrative regions: Hangzhou, Ningbo, Wenzhou, Jiaxing, Huzhou, Shaoxing, Jinhua, Quzhou, Zhoushan, Taizhou, and Lishui. The monitoring of maternal health care services has been performed since the early 1990s. In addition, a network to monitor child health care services throughout the Province has been established. However, no studies have been conducted on child health care services in Zhejiang Province.

In the present study, we investigated the levels of primary health care services for children under 7 years old and trends from 1998 to 2011, which is helpful to promote the primary health care services in our province, even the whole China.

## Subjects and Methods

### Data Collection

The data about child health care services in Zhejiang Province were extracted from the Zhejiang Maternal and Child Health Statistics collected under the supervision of the Health Bureau of Zhejiang Province. The data cover the resident population of Zhejiang Province, including both urban and rural areas. The data about the numbers of newborns and children in Zhejiang Maternal and Child Health Statistics were extracted from Household Registration Management System. The other data were reported to information collection centers in the 11 administrative regions and then reported to the Department of Maternal and Child Health Care in the Health Bureau of Zhejiang Province. All of the data were entered by two independent researchers, and there were professionals at the 11 administrative centers and the Department of Maternal and Child Health Care in the Health Bureau who looked for logical errors.

This study was approved by the Ethical Committee of the Children’s Hospital of Zhejiang University School of Medicine.

### Indicators and Definition

The indicators used in our analysis were defined by the Ministry of Health as shown in Table 1. The number of newborns every year, some health service indictors and outcome indicators were analyzed. Health service indicators included the rate of primary health care coverage for children under 3 years old and children under 7 years old, the hospital delivery rate, the low birth weight rate, the postnatal visit rate, the breastfeeding rate, and the prevalence of underweight among children under 5 years old. The mixed and breastfeeding data were collected from 2001 year while exclusive breastfeeding data were collected from 2003 from two third counties in 11 administrative centers. Outcome indicators, including early neonatal, neonatal, infant, and children under-5 mortality rates, were analyzed as well.

**Table 1:** Definitions of the indicators.

Indicator	Definition
Number of newborns	The number of newborns whose parents belong to the resident population (not the nonresident population) of Zhejiang Province every year.†
Coverage rate	The number of children who completed the regular physical examination and vaccination schedule described in the “Child Health Handbook” (per 100 children).‡
Hospital delivery rate	The number of newborns delivered in a hospital (per 100 newborns).
Low birth weight rate	The number of newborns with birth weight less than 2.5 kg (per 100 newborns).
Postnatal visit rate	The number of newborns who received a postnatal home visit at least one time during the first month by a child health care worker (per 100 newborns).
Breastfeeding rate	The number of infants who were exclusively breastfed in the first 4 months (per 100 infants).
Prevalence of underweight	The number of children whose weight is less than the mean - 2SD of the healthy reference for children of the same age and gender (per 100 children).
Early neonatal mortality	The number of newborns who died with 7 days (per 100 newborns).
Neonatal mortality	The number of newborns who died within the neonatal period (per 1000 newborns).
Infant mortality	The number of infants who died in infancy (per 1000 infants).
Under-5 mortality	The number of children who died before the age of 5 years (per 1000 children).

† In China, there is a strict household registration system. People who work in the same place where their households are registered are called the resident population, whereas people who work in a different place from where their households are registered are called the nonresident population.

‡ According to the Maternal and Infant Health Care Law of China, all newborns should be registered and given the “Child Health Care Handbook” from the medical institution in the community, village or town. The child can then receive certain primary health care services, included free regular physical examinations at set time points (3-, 6-, 9-, and 12 months old, 2 times every year from 1- to 3 years old, and one time every year from 3- to 7 years old), neonatal disease screening, and free regular vaccinations.

### Statistical Analysis

Statistical analyses were conducted using SPSS software (version 15.0). Missing data were excluded from the statistical analysis for the variable. The Pearson chi-square test was used to compare the categorical variables among different groups. Two-sided p-values were calculated, and p-values less than 0.05 were considered to indicate a significant difference.

## Results

**Figure 1:**
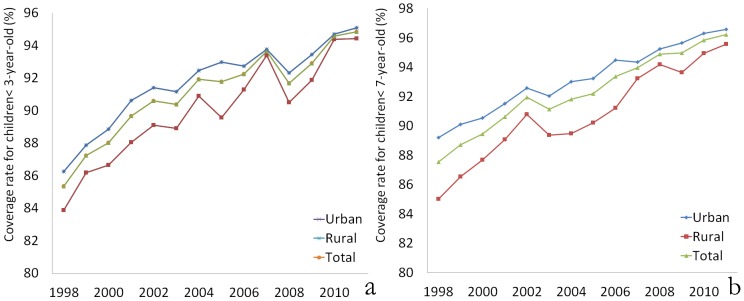
The primary health care coverage rates. (a) For children under 3 years old; (b) For children under 7 years old.

The primary health care coverage rate for children under 3 years old increased from 87.55% in 1998 to 96.22% in 2011, approximately 10 percentage points. The rates in rural areas were lower than those in urban areas each year (P<0.05). In addition, the coverage rate for children under 7 years old increased from 85.33% to 94.58% over the last 14 years. The rate in rural areas was lower than the rate in urban areas each year for this age group as well. The mean coverage rates for children under 3 years old in rural areas and urban areas were 89.82% and 91.76%, respectively, and there was a significant difference between these rates (P<0.001). The mean coverage rates among children under 7 years old in rural areas and urban areas were 90.54% and 93.18%, respectively, and there was a significant difference between these rates (P<0.001). It is notable that the difference between these two coverage rates declined in recent years (0.5–1.0%) compared to 1998 (3–5%), as shown in Figure 1.

**Figure 2:**
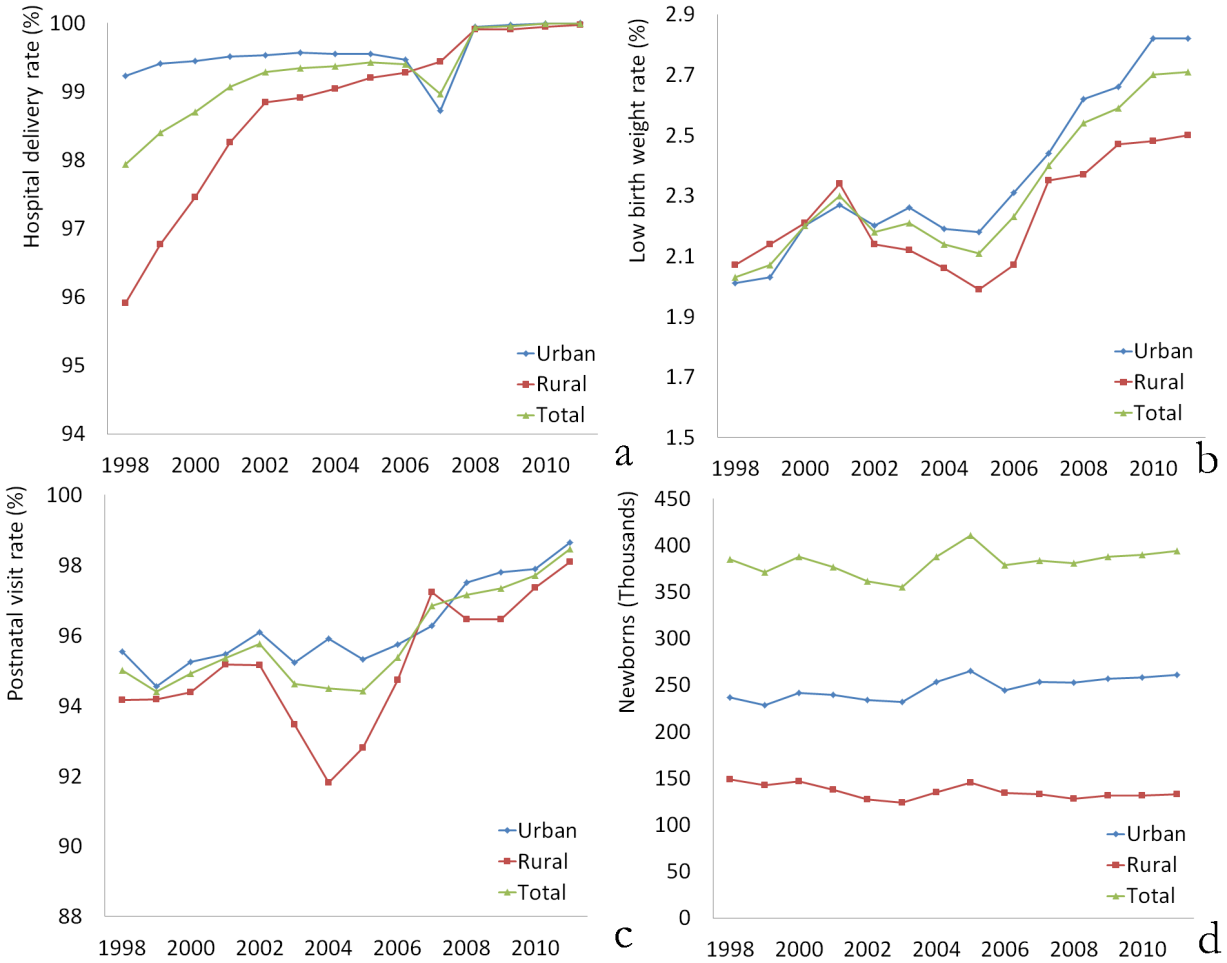
Indicators of child health and service utilization. (a) Number of newborns; (b) Hospital delivery rate; (c) Low birth weight rate; (d) Postnatal visit rates.

Over the past 14 years, 5,349,730 newborns were born in Zhejiang Province, with an average number of 382,123 every year. The number of newborns has increased in urban areas while it decreased in rural areas (Figure 2a). The ratio of the newborns born in urban and rural areas is currently about 2∶1. The hospital delivery rate was high in Zhejiang during the study period, and the overall difference narrowed substantially over the period. The rate increased slightly from 99.23% to 100.00% in urban areas, and the rate increased from 95.91% to 99.98% in rural areas. The difference in the hospital delivery rates between urban and rural areas in 2011 was very small (100.00% vs. 99.98%), as shown in Figure 2b.

It is notable that the rate of low birth weight increased during this period. The rate was significantly higher in 2011 (2.71%) than in 1998 (2.03%) (P<0.001). Moreover, the increase was more significant in urban areas (from 2.01% to 2.82%) than in rural areas (2.03% to 2.50%), as shown in Figure 2c. The postnatal visit rate was significantly lower in 1998 (95.00%) than in 2011 (98.45%) (P<0.001). It was similar or slightly higher in urban areas compared to rural areas (Figure 2d).

The types of feeding during the first 4 months of life were also investigated, beginning with 2001. The prevalence of breastfeeding and mixed feeding infants increased from 74.45% in 2001 to 90.03% in 2003. However, the prevalence of breastfeeding and mixed feeding infants has decreased since 2003–2004, although it increased slightly in the last 3 years (88.26%). In addition, the proportion of exclusively breastfeeding infants was highest in 2004 (74.79%) and has decreased since then, with the lowest rate of 53.86% in 2008. This rate rebounded in the past 3 years. We also observed that the prevalence of exclusive breastfeeding declined more substantially in urban areas (75.19% in 2003 and 61.88% in 2011) than in rural areas (71.03% in 2003 and 64.76% in 2011), as shown in Table 2.

**Table 2:** The breastfeeding rate (%) within the first 4 months and the prevalence of underweight (%) among children under 5 years old.

Year	Mixed feeding and breastfeeding			Breastfeeding			Underweight		
	Urban	Rural	Total	Urban	Rural	Total	Urban	Rural	Total
1998							1.52 (1,096,354)	1.80 (694,187)	1.63 (1,790,541)
1999							1.50 (1,071,397)	1.55 (657,270)	1.52 (1,728,667)
2000							1.39 (1,063,519)	1.53 (628,817)	1.44 (1,692,336)
2001	74.38 (190,964)	74.58 (105,238)	74.45 (296,202)				1.47 (1,069,395)	1.62 (585,684)	1.52 (1,655,079)
2002	85.37 (193,323)	85.15 (99,711)	85.30 (293,034)				1.36 (1,071,911)	1.56 (557,422)	1.43 (1,629,333)
2003	90.27 (196,573)	89.56 (99,846)	90.03 (296,419)	75.19	71.03	73.79	1.31 (1,085,517)	1.50 (541,886)	1.37 (1,627,403)
2004	89.68 (203,678)	90.53 (103,509)	89.97 (307,187)	75.71	72.96	74.79	1.22 (1,101,095)	1.41 (545,091)	1.28 (1,646,186)
2005	89.69 (218,989)	89.56 (116,289)	89.64 (335,278)	73.51	73.30	73.44	1.12 (1,126,266)	1.24 (570,002)	1.16 (1,696,268)
2006	89.67 (207,718)	86.90 (106,633)	88.73 (314,351)	71.67	70.78	71.37	1.08 (1,152,350)	1.13 (590,833)	1.09 (1,743,183)
2007	89.52 (201,020)	87.61 (106,148)	87.61 (307,168)	70.50	68.20	69.71	1.06 (1,016,591)	1.04 (600,893)	0.93(1,617,484)
2008	85.76 (190,996)	83.98 (92,371)	85.18 (283,367)	51.80	58.12	53.86	0.85 (1,186,840)	0.97 (615,261)	0.89 (1,802,101)
2009	87.23 (200,045)	86.19 (95,897)	86.89 (295,942)	56.87	62.60	58.73	0.74 (1,199,364)	0.85 (624,308)	0.78 (1,823,672)
2010	86.73 (199,317)	87.83 (96,539)	87.09 (295,856)	59.10	64.54	60.88	0.60 (1,201,037)	0.74 (650,450)	0.65 (1,851,487)
2011	88.22 (201,543)	88.35 (96,181)	88.26 (297,724)	61.88	64.76	62.81	0.61 (1,189,160)	0.72 (625,026)	0.65 (1,814,186)
Total	87.05 (2,204,166)	86.24 (1,118,362)	86.78 (3,322,528)	54.84	55.25	54.98	1.12 (15,630,796)	1.26 (8,487,130)	1.17 (24,117,926)

Underweight among children under 5 years old was also analyzed. The prevalence decreased gradually from 1.63% in 1998 to 0.65% in 2011. The prevalence rates in rural areas were lower than those in urban areas except in 2007, when the rate in urban areas (1.12%) was significantly lower than that in rural areas (1.26%) (P<0.001), as shown in Table 2.

**Figure 3:**
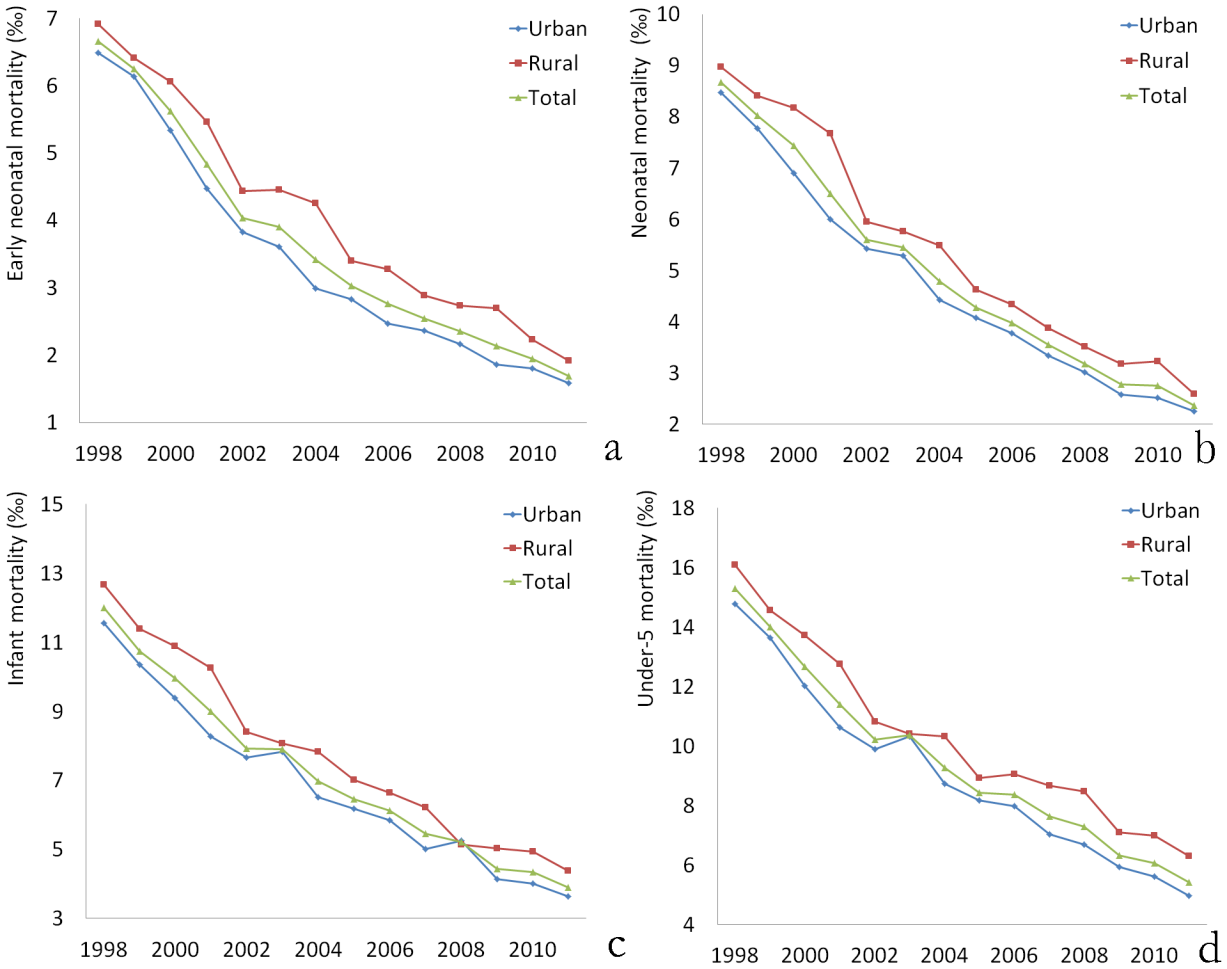
Mortality rates between 1998 and 2011. (a) Early neonatal mortality; (b) Neonatal mortality; (c) Infant mortality; (d) Under-5 mortality.

**Figure 4:**
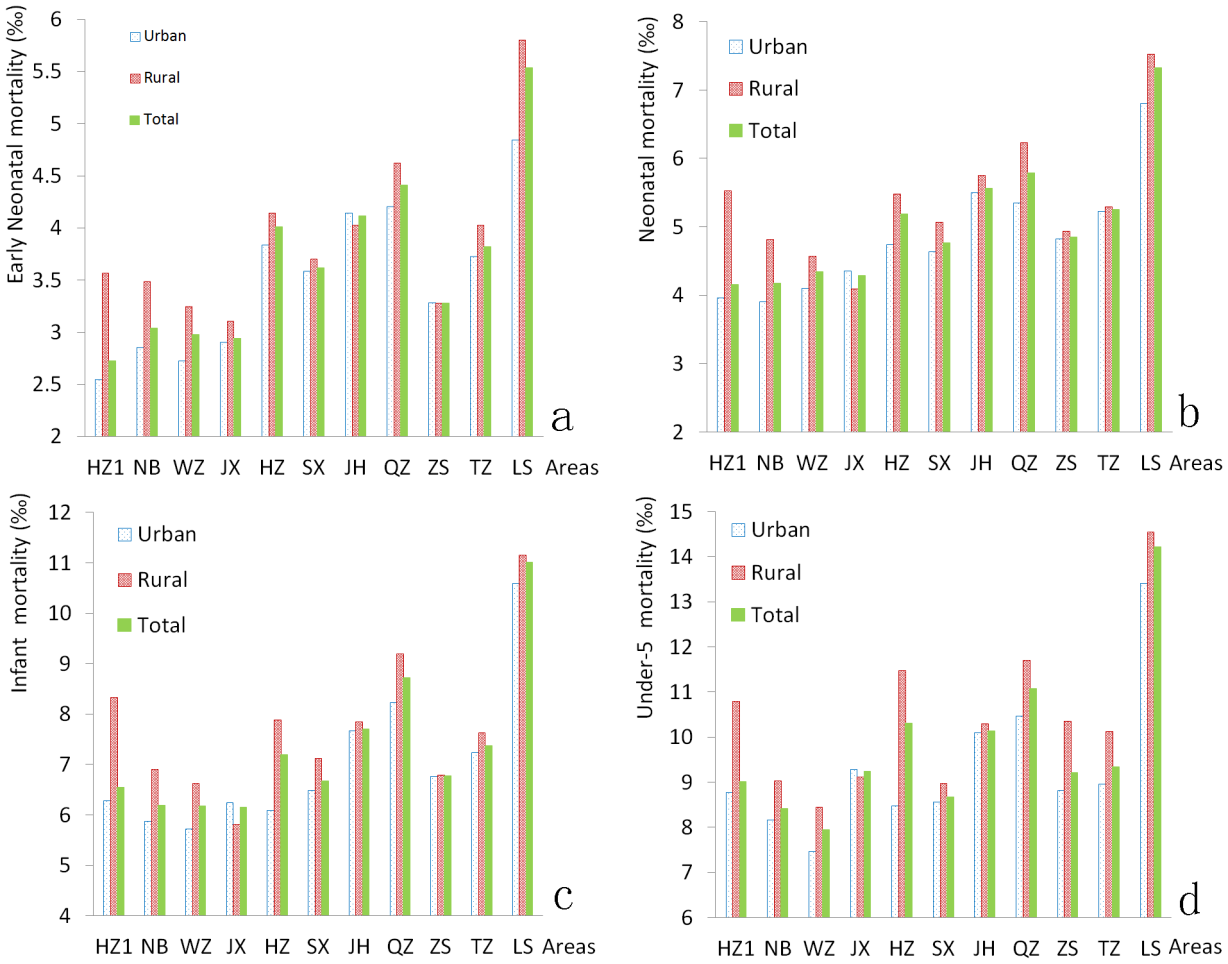
Mortality rates among the 11 administrative regions. (a) Early neonatal mortality; (b) Neonatal mortality; (c) Infant mortality; (d) Under-5 mortality. (HZ1, Hangzhou; NB, Ningbo; WZ, Wenzhou; JX, Jiaxing; HZ, Huzhou; SX, Shaoxing; JH, Jinhua; QZ, Quzhou; ZS, Zhoushan; TZ, Taizhou; LS, Lisui).

In analyzing the deaths of children under 5 years old, we noticed that over half of the deaths (26,375/50,521, 52.21%) occurred in the neonatal period, and over two-thirds of those deaths (19,478/26,375, 73.85%) occurred in the early neonatal period. During this period, mortality rates decreased dramatically. Early neonatal mortality decreased significantly from 6.66‰ in 1998 to 1.69‰ in 2011 (P<0.001). Neonatal mortality decreased from 8.67‰ in 1998 to 2.36‰ in 2011. Infant mortality decreased from 11.99‰ in 1998 to 3.89‰ in 2011. Under-5 mortality decreased from 15.28‰ in 1998 to 5.42‰ in 2011. These 4 types of mortality rates were all slightly higher in rural areas than in urban areas each year (Figure 3). In addition, we have analyzed the mortality rates according to region. We noted that the mortality rates were lower in Ningbo, Wenzhou, and Jiaxing regions and higher in Quzhou and Lishui regions (Figure 4).

## Discussion

As social and economic development progresses, more and more attention has been focused on maternal and child health, especially on reducing maternal mortality and mortality among children under 5 years old. Since the early 1990s, networks of maternal and child health care services have been established and monitored in Zhejiang Province. These networks currently cover over 99% of the provincial population, including part of the nonresident population. The network coverage rate in Zhejiang is higher than the average level in China (75% of children under 7 years old in 2005) [7]. Furthermore, neonatal screenings for phenylketonuria and congenital hypothyroidism have been performed since 1999 and are now offered to over 97% of newborns in Zhejiang Province [8], [9]. The TORCH panel and diagnostic screenings for human immunodeficiency virus, syphilis, antenatal conditions, and anemia of the mother and child have been performed since 2008 (data not shown), in accordance with the WHO recommendations [10].

During the past 14 years, there have been significant improvements in child health care, including prenatal care, delivery care, and postnatal care. The primary health care coverage rates for children under 3 -years old and children under 7 years old increased approximately 10%, although internal fluctuation and variations were observed. Moreover, the difference between rural and urban areas narrowed in recent years. These improvements may be attributed to general socioeconomic development, including the increase in income, living standards, and educational levels [11]–[13], and government initiatives, such as new laws, increased funding for health care, neonatal screenings, and a nationwide free vaccination program [7], [14], [15].

The coverage rates for children under 3 -years old and children under 7 -years old were lower in rural areas than in urban areas during the study period, although the difference was slight. This result is similar to other reports from other areas of China and other developing countries and may be associated with issues of geographical distance, socioeconomic level, education level, and health awareness [13], [16], [17].

Although the number of newborns was relatively stable over the 14-year period in the present study, there was a slight increase in urban areas and a slight decrease in rural areas. Currently, the ratio of newborns born in urban areas versus rural areas is approximately 2∶1. This figure is correlated with the changes in the number of women of childbearing age, which may be associated with the urbanization of China since the reforms in the 1980s. This tendency has also been seen in other developing countries [18].

Both the hospital delivery rate and the postnatal home visit rate were high in Zhejiang Province. The rate of low birth weight among newborns also increased during these years, especially in urban areas. The data seem to contrast with socioeconomic development and improvements in health care. However, the rate of high-risk pregnancies also tended to increase during this period. The high-risk pregnancy rate in 2010 was approximately 2 times the rate in 1998 (data not shown). This result may be associated with two facts. First, with improvements in health care and better interventions, some fetuses with defects may survive now, whereas they may have been aborted or died in the past [19], [20]. Second, some adverse factors increased because of environmental factors, lifestyle habits, and work stress, which are associated with the excessive emphasis on economic development. In addition, we found a higher rate of high-risk pregnancies in urban areas than in rural areas, which may be related to the work stress, activities, and medical technology in urban areas.

Feeding practices were not assessed in Zhejiang Province until 2001. This study revealed that the rate of breastfeeding reached its peak in 2003 and 2004 and then declined. Although the rate recovered after 2008, the rate of mixed feeding and breastfeeding within the first 4 months still did not reach 90%, and the exclusive breastfeeding rate was only 62.81% in 2011. Moreover, we noted that the exclusive breastfeeding rate in urban areas was higher than the rate in rural areas from 2003 to 2007, but the pattern reversed in 2007. This change may reflect the economic pressure on young parents (e.g., returning to work within 4 months after delivery, developing breast issues because of various pressures), especially parents in urban areas.

Although children’s heights were not recorded in the Zhejiang maternal and child health statistics, the prevalence of underweight among children under 5 years old partially reflects the nutrition of these children after birth. We found that the prevalence of underweight decreased to 0.65% in 2011. However, the number of overweight and obese children has dramatically increased in recent years. It has been reported that more than 10% of school-aged children and adolescents were overweight and obese in Taijin [21], with similar statistics in 6 other provincial capital cities [22]. Therefore, more attention should be given to both ends of the weight spectrum: underweight and overweight.

By analyzing the deaths of children under 5 years old, we noted that over half of the deaths occurred during the neonatal period, and over two-thirds of those deaths occurred in the early neonatal period. This result suggests that improving the newborn survival rate, especially in the first week after birth, is important for reducing under-5 mortality. This requires the efforts of obstetricians to reduce perverse labor and pediatricians to elevate the remedy ability.

It is encouraging that mortality rates have decreased dramatically over the past 14 years. Early neonatal mortality decreased approximately 5‰ from 1998 to 2011 (from 6.66‰ to 1.69‰). The neonatal, infant, and under-5 mortality rates were approximately 6‰, 8‰, and 10‰, respectively. The mortality rates are lower than those in most developing countries [23], [24] (although they are higher than those in some developed countries), indicating that the government efforts in health care have resulted in excellent achievement in recent years [25].Mortality rates in rural areas were slightly higher than the rates in urban areas. Mortality was lower in Ningbo, Wenzhou and Jiaxing regions and higher in Quzhou and Lishui regions, where the socio-economic level is lower. The difference may be associated with the lack of health awareness and poor medical technology in rural areas [26], [27], suggesting that more attention should be given to primary health services for children in rural areas and regions with lower socio-economic levels.

There were several limitations to this study. First, we did not check the raw data directly because this dataset was so large. Second, some children may not be included in these data because of the family planning policy (i.e., it is illegal to have more than two children for most parents) and adoption without lawful procedures, although there is a strict household registration management system in China. Third, it is possible that deaths were under-reported and postnatal visits were over-reported because of the desire to demonstrate the achievements of local government, limited willingness to participate, or poor capacity to track high-risk infants, critically ill infants, and/or self-discharged patients.

In summary, primary health care services for children in Zhejiang Province improved from 1998 to 2011. However, some factors that may affect child health care utilization, such as health awareness and medical technology, still need improvement.
